# Risk Factors and Disability Associated with Low Back Pain in Older Adults in Low- and Middle-Income Countries. Results from the WHO Study on Global AGEing and Adult Health (SAGE)

**DOI:** 10.1371/journal.pone.0127880

**Published:** 2015-06-04

**Authors:** Jennifer Stewart Williams, Nawi Ng, Karl Peltzer, Alfred Yawson, Richard Biritwum, Tamara Maximova, Fan Wu, Perianayagam Arokiasamy, Paul Kowal, Somnath Chatterji

**Affiliations:** 1 Department Public Health and Clinical Medicine Epidemiology and Global Health, Umeå University, Umeå, Sweden; 2 Research Centre for Gender, Health and Ageing, Faculty of Health, University of Newcastle, Newcastle, Australia; 3 Human Sciences Research Council, Pretoria, South Africa; 4 Department of Psychology, University of the Free State, Bloemfontein, South Africa; 5 ASEAN Institute for Health Development, Mahidol University, Nakhon Pathom, Thailand; 6 Department of Community Health, University of Ghana, Accra, Ghana; 7 National Research Institute of Public Health (FSBI, RAMS), Moscow, Russian Federation; 8 Shanghai Municipal Center for Disease Control and Prevention, Shanghai, China; 9 International Institute of Population Sciences, Deonar, Mumbai, India; 10 World Health Organization Study on global AGEing and adult health, Geneva, Switzerland; 11 Surveys, Measurement and Analysis Unit, World Health Organization, Geneva, Switzerland; Karolinska Institutet, ITALY

## Abstract

**Background:**

Back pain is a common disabling chronic condition that burdens individuals, families and societies. Epidemiological evidence, mainly from high-income countries, shows positive association between back pain prevalence and older age. There is an urgent need for accurate epidemiological data on back pain in adult populations in low- and middle-income countries (LMICs) where populations are ageing rapidly. The objectives of this study are to: measure the prevalence of back pain; identify risk factors and determinants associated with back pain, and describe association between back pain and disability in adults aged 50 years and older, in six LMICs from different regions of the world. The findings provide insights into country-level differences in self-reported back pain and disability in a group of socially, culturally, economically and geographically diverse LMICs.

**Methods:**

Standardized national survey data collected from adults (50 years and older) participating in the World Health Organization (WHO) Study on global AGEing and adult health (SAGE) were analysed. The weighted sample (n = 30, 146) comprised respondents in China, Ghana, India, Mexico, South Africa and the Russian Federation. Multivariable regressions describe factors associated with back pain prevalence and intensity, and back pain as a determinant of disability.

**Results:**

Prevalence was highest in the Russian Federation (56%) and lowest in China (22%). In the pooled multi-country analyses, female sex, lower education, lower wealth and multiple chronic morbidities were significant in association with past-month back pain (p<0.01). About 8% of respondents reported that they experienced intense back pain in the previous month.

**Conclusions:**

Evidence on back pain and its impact on disability is needed in developing countries so that governments can invest in cost-effective education and rehabilitation to reduce the growing social and economic burden imposed by this disabling condition.

## Introduction

Back pain is a highly prevalent disabling musculoskeletal condition affecting almost everyone at some time [1]. The biopsychosocial model is the prevailing framework used for understanding, managing and treating back pain. This approach suggests that in addition to biology, psychological, socio-economic, environmental and cultural factors all contribute to the incidence and persistence of back pain symptoms [2, 3]. Many musculoskeletal conditions start in middle-age and require interactions with health care providers over many years [1, 4, 5]. Low back pain, or “back pain”, is a leading cause of activity limitation, work absenteeism and lost productivity throughout much of the industrialized world–threatening function, mental health and quality of life [6–8] and inflicting substantial direct and indirect costs on health, social and economic systems [1].

Globally back pain causes more disability than any other condition. The 2010 Global Burden of Disease Study ranked low back pain as the condition with the highest number of years lived with disability (YLDs) and sixth in terms of disability-adjusted life years (DALYs) [8, 9]. In 1990, the global burden of YLDs due to back pain in adults aged 50–69 was 59% in developing countries, but by 2010 this proportion had increased to 67% [10]. With rapid growth in the numbers and proportions of older adults in low- and middle-income countries (LMICs) the back pain burden in older adults in these countries is expected to grow significantly in coming decades [1, 8, 9, 11].

Back pain is also one of the most common conditions for which patients in high-income countries seek medical care [12]. Most of the information about back pain has come from developed countries in Europe, North American and Australasia, making it difficult to draw comparisons with developing countries [12–15]. Italian researchers reported back pain prevalence of 32% in adults aged over 65 years [16] and a study of community-dwelling adults aged 70–79 years in the United States (US) demonstrated back pain prevalence of 36%. A review of the prevalence of musculoskeletal conditions in adults aged 60 and over in developed countries reported one-month back pain prevalence of between 18% and 29% [17].

The Jerusalem Longitudinal study [18] showed that chronic back pain was prevalent in the elderly (aged 70 years and over) and that psychosocial factors, female gender and hypertension were associated with back pain. Association between back pain and older age is also heavily modified by the severity and intensity of the complaint [19]. Studies conducted in North America, [20, 21] Europe, [12, 22, 23] and Australasia [17] found that the prevalence and intensity of back pain is associated with individual, psychosocial and occupational factors. In addition to being older and female [1, 24, 25] modifiable determinants of back pain in developed countries include smoking, depression, lack of physical activity and abdominal obesity [12, 23, 24, 26–30]. A Japanese study of men aged 40 years and older demonstrated that back pain had a significant negative impact on quality of life [31]. European studies provide evidence of inverse association between back pain and socioeconomic factors, such as older age, higher income and education [32–35].

Although a few studies investigating the determinants of back pain have been conducted in developing countries, the literature is sparse compared with developed countries. In a community-based study of adults in Korea (mean age 56 years) the common determinants were advancing age and female sex [36]. Studies conducted in Taiwan [37], China [38] and Sri Lanka [39] have focused on working-age populations. A review of back pain prevalence studies conducted in Sub-Saharan Africa on mostly working-age adults and adolescents, concluded that back pain prevalence was rising [25].

The perception and reporting of back pain is influenced by individual characteristics, working conditions, lifestyle, and social, economic, cultural and ethnic factors, as well as the availability of treatment and rehabilitation options [1, 5]. In some societies and countries there is a greater awareness of the symptoms and also a greater willingness to report them, while in others, back pain is not necessarily associated with disability, but rather seen as a natural consequence of routine physical work or the ageing process itself [1]. Given the fundamental social, cultural and economic differences between developed and developing countries, it is reasonable to argue that the antecedents and consequences of back pain are not homogeneous. For example, extreme poverty, infectious diseases epidemics, work tasks, family structures, responsibilities, social expectations, geography, health care availability and support may all impact differently on the perception and reporting of back pain in different contexts and settings.

As a consequence of the major epidemiological and demographic transitions occurring in emerging economies in all regions of the world, there is now an urgent need to gather accurate comparable epidemiological data on back pain in older adult populations in developing countries [11, 29, 36, 40–43]. This research draws attention to the complexity of predisposing, enabling and contextual factors that require consideration at the country level [5].

According to the World Health Organization (WHO) one of the most disabling conditions among the elderly is musculoskeletal disorders, of which back pain is a major contributor [8, 14, 17, 40, 43]. Data collected from adults aged 50 years and older participating in the WHO Study on global AGEing and adult health (SAGE) were analysed to: measure the prevalence of back pain; identify risk factors and determinants associated with back pain prevalence and intensity, and describe association between back pain and disability. The purpose is to gain insights into country-level differences in self-reported back pain in a group of socially, culturally, economically and geographically diverse LMICs.

## Methods

### Ethics Statement

The SAGE study was approved by the following bodies: the Ethics Review Committee, World Health Organization; Ethical Committee, Ghana Medical School, Accra, Ghana; Ethics Committee, OPM (School of Preventive and Social Medicine), Russian Academy of Medical Sciences, Moscow, Russia; Ethics Committee, Shanghai Municipal Centre for Disease Control and Prevention, Shanghai, China; Institutional Review Board, International Institute of Population Sciences, Mumbai, India; Research Ethics Committee, Human Sciences Research Council, Pretoria, South Africa; and Ethics Committee, National Institute of Public Health (INSP), Cuernavaca, Mexico. This approval also covered all procedures by which written informed consent was obtained from each individual participant. Confidential records of participants’ consent were maintained by SAGE.

### Data Collection

The SAGE Wave 1 is a longitudinal study that provides the baseline round of data for nationally representative samples of adults aged 50 years and over in China, Ghana, India, Mexico, the Russian Federation and South Africa. Cross sectional data from SAGE Wave 1 were collected via in-person structured interviews (2007–2010). All six SAGE countries implemented multistage cluster sampling strategies [44]. Household-level and person-level analysis weights, based on the selection probability at each stage of sampling along with post stratification corrections, were applied to produce nationally representative cohorts. Age and sex standardizations based on WHO’s World Standard Population [45] and the United Nations Statistical Division’s population distributions (http://unstats.un.org/unsd/default.htm)) were carried out to adjust for between country population age and sex differences. Additional details about SAGE are provided elsewhere [46].

### Sample

The sample in this study included adults aged 50 years and over in the six SAGE countries. There were 29, 807 observations: 11,648 in China; 4,072 in Ghana; 6,350 in India; 2,004 in Mexico; 2,933 in the Russian Federation and 2,805 in South Africa. Weighted samples were 30,146 for the six countries comprising: 11,525 in China; 4,059 in Ghana; 6,329 in India; 2,973 in the Russian Federation and 2,720 in South Africa.

### Variables

#### Dependent Variables

Three dependent variables–past-month back pain prevalence, back pain frequency/intensity and disability—were derived from questions in the SAGE individual questionnaire.

Past-month back pain prevalence (no vs. yes) was identified from responses to the question “Have you experienced back pain in the last 30 days?”

A score measuring the intensity and frequency of past-month back pain, was conditioned on responses (yes) to this prevalence question (4008). Frequency was measured using responses to question 4009 which asked “On how many days did you have this back pain during the last 30 days?” A pain intensity measure was derived by summing responses to questions 2007 and 2008. In these two questions respondents were asked to use a Likert scale (1 = none, 2 = mild, 3 = moderate, 4 = severe and 5 = extreme) to rate the extent of overall bodily pain (question 2007) and discomfort (question 2008) experienced in the previous 30 days. The intensity and frequency score was computed as the product of these two measures. This was converted to a percentile index, with zero and 100 indicating the minimum and maximum possible scores. The distribution was skewed (skewness = 1.48, median 8.3, mean = 18.2 and standard deviation = 21.6). Scores were grouped into three categories: low (zero score) vs. moderate (> = 50) vs. high intensity (>50). The proportion of respondents in the categories varied across countries ranging from: 9% to 22% in the low group, 72% to 82% in the moderate group and 4% to 12% in the high group. This intensity/frequency variable was used here as the measure of “back pain intensity”.

Disability was measured using the WHO Disability Assessment Schedule (WHODAS 2.0) encompassing six domains [47, 48]. Twelve items were included in the scale asking about difficulty in functioning in the past 30 days. This included activities of daily living (such as, standing, dressing oneself) and instrumental activities of daily living (such as learning a new task, participating in community activities, household chores). Responses were measured on a Likert scale, ranging from no difficulty to severe difficulty or cannot perform the activity, and summed to a composite score which was transformed to a scale of 0–100, with 100 indicating the most severe disability [48].

#### Socio-demographic variables

Data on socio-demographic characteristics collected in SAGE were used to describe the study sample. The variables were sex: male vs. female; age: 50–59 years vs. 60–69 years vs. 70–79 years vs. 80-plus years; education level: no primary completed vs. completed primary vs. completed secondary or high school vs. completed university or college; marital status: never married, vs. married/cohabiting vs. divorced/separated/widowed; work status: never worked vs. currently working vs. not currently working; wealth quintiles: 1 (poorest) to 5 (richest) and area of residence: urban vs. rural. A random-effects probit model (previously developed and reported elsewhere) was used to estimate wealth levels based on asset ownership [49, 50]. This was applied to every household in the SAGE surveys and used to establish country-specific quintiles of household wealth made available by the WHO. The quintiles provide an alternative measure of income and assets that is less likely to be biased by contextual differences than traditional income-based measures.

#### Health-related variables

Health-related variables are described here. Responses to questions on the use of alcohol and tobacco were categorised as smoker: not current vs. current; and alcohol: never drinkers vs. former drinkers vs. current drinkers. Obesity was measured using waist circumference rather than body mass index (BMI). In making this decision we considered evidence of the importance of waist circumference as a predictor of health outcomes [30, 51–53] and the availability of WHO recommendations for waist circumference in men and women separately [54]. The waist circumference variable (low risk vs. high risk) was derived using WHO recommended cut-offs of > = 102 centimetres for men and > = 88 centimetres for women.

Physical activity was measured using the Global Physical Activity Questionnaire (GPAQ) [55, 56] which collects information on sedentary behaviour and physical activity participation in work, travel, and recreation. A categorical variable measuring low vs. moderate vs. high physical activity was included. High physical activity was defined as vigorous-intensity activity (such as running which increases the heart rate and breathing) on at least three days per week, or seven or more days per week of any combination of walking, moderate or vigorous intensity activities. Moderate physical activity was defined as (per week) three or more days of vigorous-intensity activity, or five or more days of moderate-intensity activity (such as walking or cycling resulting in a small but noticeable increase in the heart rate and breathing) of at least 30 minutes per day, or five or more days of any combination of walking, moderate or vigorous intensity activities. Low physical activity was defined as not meeting any of these criteria.

Indicator variables (no vs. yes) for symptom-based arthritis, depression, asthma [57, 58] and self-reported diabetes mellitus are included. Symptom-based conditions were derived using validated WHO algorithms. A “chronic count” variable (zero vs. one vs. two or more chronic conditions) was included.

### Statistical analyses

Data presented here are weighted and include post stratification adjustments in national country samples and in the pooled multi-country data set [45].

We undertook a complete case analysis. Data were missing for: education (2.2%); marital status (0.6%); work status (1.4%); wealth (0.4%); smoking (1.9%): drinking (2.1%); waist circumference (7.0%); physical activity (1.6%); depression (2.1%); arthritis (2.2%); asthma (2.2%); diabetes (1.8%) and the count of chronic conditions (1.7%).

Descriptive statistics for socio-demographic variables are presented as proportions for each country and pooled. The prevalence of back pain and back pain intensity (conditioned on prevalence) is shown by countries and pooled, and also by socio-demographic and health-related characteristics in the pooled sample.

Three sets of multivariable regression were undertaken using the pooled multi-country data set. Multivariable logistic regression describes association between socio-demographic and health-related determinants and back pain prevalence. Multivariable ordinal logistic regression describes association between socio-demographic and health-related determinants and back pain intensity (low vs. moderate vs. high). Multivariable linear regression describes association between back pain intensity (independent variable) and disability (dependent variable) adjusting for confounding by socio-demographic and health-related factors. A country variable (reference China) was included in all multivariable regressions.

The literature includes a substantial number of factors associated with back pain and disability. In aiming to achieve relatively parsimonious models, we focused on recurrent, commonly cited factors, identifiable in the SAGE data. These factors were tested in bivariate analyses, and where statistically significant (p<0.05), were included as covariates in the multivariable regressions.

Odds ratios and 95% confidence intervals are reported. Variables were tested for correlation and multicollinearity. Diagnostic checks were undertaken on models and no violations of assumptions were found. STATA Version 11 (StataCorp, 2009) was used for all statistical analyses.

## Results

Back pain prevalence was estimated on the weighted sample of 30,146 derived from 29,807 individual observations in the six SAGE countries pooled. In order to measure back pain intensity, the analysis was conditioned on back pain prevalence, giving a sub-sample of 8,815.

Table 1 compares socio-demographic characteristics of the study sample by countries and pooled (n = 30,146). There were more females in the study population except in Ghana and India. In most countries, almost half of the study respondents were aged between 50–59 years. In contrast to Ghana and India, where over 60% of the respondents reported no primary education, almost 18% of respondents in the Russian Federation reported that they had completed university or college education. The majority of the respondents were married or cohabiting at the time of the survey. Almost 40% of the respondents were separated, divorced or widowed in Ghana and the Russian Federation. Over 56% reported that they were currently working in the Russian Federation compared with 23% in Mexico. About 72% of respondents in India lived in rural areas, compared with only 22% and 30% in Mexico and the Russian Federation respectively.

**Table 1:** Weighted proportional distribution of socio-demographic characteristics, adults aged 50-plus years, by country and pooled countries, SAGE Wave 1.

	China	Ghana	India	Mexico	Russian Fed^a^	Sth Africa^b^	Pooled
**Weighted Sample**	11,525	4,059	6,329	2,103	2,973	2,720	30,146
**Observations**	11,648	4,072	6,350	2,004	2,933	2,805	29,807
**Sex (%)**							
Male	49.2	52.5	51.2	46.0	37.8	40.1	48.5
Female	50.8	47.5	48.8	54.0	62.2	59.9	51.5
**Age Group (%)**	****						
50–59 years	45.3	40.2	49.1	50.6	47.5	50.2	50.2
60–69 years	31.9	27.4	30.9	25.7	25.2	30.2	28.8
70–79 years	18.6	22.9	15.6	17.0	20.2	14.1	15.9
80+ years	4.2	9.5	4.3	6.7	7.1	5.5	5.1
**Education (%)**							
No primary comp^c^	42.8	63.9	61.1	55.5	1.9	49.4	46.0
Comp^c^ primary	21.7	11.1	14.7	24.2	5.0	22.0	18.4
Comp^c^ secondary/high	31.7	21.4	19.1	12.6	75.4	22.6	30.5
Comp^c^ univ^d^/college	3.8	3.7	5.2	7.8	17.7	6.0	5.1
**Marital Status (%)**							
Never married	1.0	1.2	0.7	7.2	2.4	14.7	1.3
Married/cohabiting	85.2	59.4	77.5	74.1	58.5	52.6	80.5
Separated/div^e^/widowed	13.9	39.3	21.8	18.7	39.1	32.7	18.2
**Work Status (%)**							
Never worked	43.8	70.1	43.9	38.7	42.9	31.0	45.0
Currently working	47.4	28.4	29.2	23.0	56.4	55.3	40.4
Not currently working	8.8	1.5	26.9	38.3	0.8	13.7	14.6
**Wealth Quintile (%)**							
Lowest (poorest)	16.7	18.1	18.3	15.0	14.4	19.8	17.0
Second	18.9	19.2	19.0	25.5	16.4	20.7	18.9
Third	20.4	20.5	18.9	16.3	19.3	19.9	19.6
Fourth	23.2	20.9	19.8	16.2	23.0	19.5	22.0
Highest (wealthiest)	20.7	21.4	24.0	27.1	26.9	20.1	22.5
**Residence (%)**							
Urban	46.8	41.0	28.2	78.0	70.5	64.8	42.7
Rural	53.2	59.0	71.8	22.0	29.5	35.2	57.3

^a^Russian Federation

^b^South Africa

^c^Completed

^d^University

^e^Divorced

Table 2 presents past-month prevalence (n = 30,146) and back pain intensity conditioned on prevalence (n = 8,815) for respondents 50 years and older in the six SAGE countries. Overall, the self-reported prevalence of back pain in the past month was 30%. Prevalence was highest in the Russian Federation (56%) and lowest in China (22%).

**Table 2:** Back pain prevalence and intensity, adults aged 50-plus years, by country and pooled, SAGE Wave 1.

	Back Pain Prevalence		Back Pain Intensity			
	n	% (95% CI^e^)	n	Low	Moderate	High
**China**	11,525	22.0 (19.9,24.1)	2,490	18.5 (16.5,20.7)	77.4 (75.7,79.1)	4.1 (2.5,6.5)
**Ghana**	4,059	40.5 (38.1,42.8)	1,622	8.8 (7.0,11.0)	83.1 (80.5,85.4)	8.1 (6.4,10.2)
**India**	6,329	39.1 (36.6,41.7)	2,466	8.8 (7.4,10.5)	79.0 (76.7,81.1)	12.2 (10.5,14.2)
**Mexico**	2,103	35.5 (28.5,43.1)	746	22.0 (14.9,31.1)	74.2 (64.8,81.8)	3.9 (1.8,8.1)
**Russian Fed** ^**a**^	2,973	55.7 (50.2,61.2)	1,565	19.9 (15.3,23.5)	72.0 (66.8,76.6)	8.1 (5.5,11.8)
**Sth Africa** ^**b**^	2,720	38.5 (34.9,42.2)	1,019	13.5 (10.4,17.3)	82.3 (78.3,85.6)	4.3 (3.0,6.0)
**Pooled**	30,146	29.7 (28.1,31.4)	8,815	14.9 (13.6,16.2)	77.2 (75.8,78.5)	7.9 (6.9,9.2)

^a^Russian Federation

^b^South Africa

^e^Confidence Interval

Intensity sample conditioned on prevalence

Comparing the proportion of respondents in each intensity group, India had the highest proportion of respondents in the high intensity group (12%) and China and South Africa had the lowest (4%). In the pooled analysis, 8% of respondents were in the high intensity group, compared with 77% and 15% in the moderate and low intensity groups respectively. Mexico had the highest prevalence in the low intensity group (22%) and Ghana and India had the lowest (9%).

Table 3 shows the pooled prevalence of back pain in the past month by socio-demographic and health-related characteristics (n = 30,146). Past-month back pain prevalence was high for females (35%), rural dwellers (32%) and those with high risk waist circumference (36%). Prevalence was 52% for respondents with arthritis, 55% for depression, 52% for asthma and 31% for diabetes. Amongst respondents with two or more chronic conditions, the prevalence of back pain was 59%. There are prevalence gradients for wealth, education and age, with higher wealth and higher education associated with lower prevalence, and older age associated with higher prevalence.

**Table 3:** Back pain prevalence and intensity by socio-demographic and health characteristics, adults aged 50-plus years, pooled countries SAGE Wave 1.

	Back Pain Prevalence	Back Pain Intensity % (95% CI^a^)		
	% (95% CI^a^)	Low	Moderate	High
**Observations**	30,146	1,311	6,804	700
**Sex**				
Male	24.2 (22.5,26.1)	17.3 (15.4,19.5)	77.1 (75.1,78.9)	5.6 (4.4,7.1)
Female	34.9 (33.0,36.9)	13.3 (11.9,14.8)	77.3 (75.4,79.0)	9.4 (8.1,11.0)
**Age Group**				
50–59 years	26.3 (24.5,28.3)	19.8 (17.8,21.9)	75.3 (73.0,77.5)	4.9 (3.8,6.2)
60–69 years	31.8 (29.7,34.0)	13.6 (11.8,15.7)	77.8 (75.5,80.0)	8.6 (6.7,10.8)
70–79 years	35.1 (32.6,37.7)	8.7 (7.1,10.6)	81.5 (78.3,84.3)	9.8 (7.7,12.5)
80+ years	34.9 (31.1,39.0)	5.1 (3.4,7.5)	73.8 (67.1,80.0)	21.1 (15.6,28.0)
**Education**				
No primary comp^b^	34.9 (30.0,36.9)	10.1 (8.8,11.6)	79.8 (78.0,81.6)	10.1 (8.5,11.8)
Comp^b^ primary	24.7 (22.2,27.4)	17.4 (14.6,20.5)	76.1 (72.4,79.4)	6.5 (4.9,8.7)
Comp^b^ secondary/high	26.4 (24.1,28.8)	21.6 (18.9,24.5)	73.5 (70.5,76.2)	5.0 (3.7,6.7)
Comp^b^ univ^c^/college	21.2 (17.8,25.0)	26.6 (20.6,33.7)	69.4 (62.5,75.5)	4.0 (2.2,7.2)
**Marital Status**				
Never married	23.7 (19.0,29.1)	19.3 (12.3,28.8)	77.4 (66.8,85.3)	3.4 (1.4,8.0)
Married/cohabiting	27.7 (26.0,29.4)	16.3 (14.8,18.0)	76.9 (75.4,78.3)	6.8 (5.7,8.0)
Separated/div^d^/widowed	39.3 (36.9,42.0)	10.1 (8.6,11.8)	78.0 (75.3,80.5)	11.9 (9.8,14.4)
**Work Status**				
Never worked	26.9 (24.6,29.2)	18.2 (16.1,20.5)	77.0 (74.9,79.0)	4.8 (3.6,6.4)
Currently working	30.6 (28.6,32.8)	13.9 (12.2,15.8)	76.6 (74.4,78.6)	9.5 (8.0,11.4)
Not currently working	36.2 (33.2,39.3)	9.8 (7.9,12.0)	80.0 (75.6,82.0)	11.3 (9.0,14.0)
**Wealth Quintile**				
Lowest (poorest)	36.2 (33.5,38.9)	10.7 (8.9,12.7)	81.0 (78.4,83.3)	8.4 (6.7,10.3)
Second	31.5 (29.1,34.0)	13.2 (11.2,15.5)	77.2 (74.0,80.1)	9.6 (7.1,12.9)
Third	30.8 (27.8,34.0)	13.6 (11.6,15.8)	78.5 (75.5,81.2)	8.0 (6.2,10.2)
Fourth	27.4 (24.8,30.2)	18.4 (15.7,21.5)	73.6 (70.3,76.6)	8.0 (6.0,10.6)
Highest (wealthiest)	24.7 (21.7,28.O)	19.1 (15.7,23.1)	75.4 (71.6,78.8)	5.5 (4.0,7.6)
**Residence**				
Urban	27.4 (25.2,30.0)	19.6 (17.2,22.2)	74.8 (72.6,76.9)	5.6 (4.3,7.2)
Rural	31.5 (29.2,33.8)	11.8 (10.5,13.2)	78.7 (77.1,80.3)	9.5 (8.0,11.3)
**Smoker**				
Not current	29.6 (27.9,31.4)	15.4 (13.9,17.1)	76.9 (75.1,78.7)	7.7 (6.4,9.1)
Current	30.0 (27.9,31.1)	13.7 (11.6,16.1)	77.7 (75.2,80.1)	8.6 (6.9,10.6)
**Alcohol**				
Never drinkers	29.7 (28.0,31.4)	13.7 (12.3,15.4)	77.1 (75.3,78.7)	9.2(7.9,10.8)
Former drinkers	34.5 (31.2,37.9)	14.9 (12.2,18.0)	79.0 (75.6,82.0)	6.1 (4.7,7.9)
Current drinkers	26.8 (23.9,29.4)	19.4 (16.6,22.6)	76.2 (73.3,78.8)	4.5 (3.3,6.1)
**Waist Circumference**				
Low risk	28.0 (26.3,29.6)	15.1 (13.7,16.6)	77.0 (75.5,78.5)	7.9 (6.7,9.3)
High risk	35.9 (33.6,38.3)	14.2 (12.0,16.6)	77.7 (75.0,80.1)	8.1 (6.6,10.0)
**Physical Activity**				
High	30.9 (28.9,33.1)	16.2 (14.5,14.2)	78.2 (76.4,79.8)	5.7 (4.6,6.9)
Moderate	27.7 (25.5,30.09	15.2 (12.8,17.9)	77.6 (74.7,80.3)	7.2 (5.6,9.3)
Low	29.4 (27.1,31.9)	12.1 (10.1,14.4)	75.0 (72.0,77.7)	13.0 (10.6,15.8)
**Arthritis**				
No	23.5 (22.0,25.0)	18.2 (16.4,20.1)	74.9 (73.0,76.7)	6.9 (5.9,8.1)
Yes	51.9 (48.8,54.9)	9.5 (8.1,11.1)	80.9 (78.5,83.1)	9.6 (7.8,11.8)
**Depression**				
No	27.6 (26.0,29.2)	16.6 (15.2,18.2)	77.7 (76.2,79.1)	5.7 (4.8,6.9)
Yes	55.1 (49.4,60.8)	4.9 (3.6,6.6)	74.5 (70.0,78.5)	20.6 (16.9,24.9)
**Asthma**				
No	28.3 (26.7,29.9)	16.1 (14.8,17.6)	76.6 (75.3,78.0)	7.2 (6.2,8.4)
Yes	52.4 (48.3,56.4)	6.3 (4.4,8.9)	76.0 (70.7,80.5)	17.8 (13.4,23.3)
**Diabetes**				
No	29.6 (28.0,31.3)	15.1 (13.8,16.5)	77.Q (75.7,78.4)	7.8 (6.8,9.0)
Yes	31.4 (27.5,35.6)	11.9 (8.8,15.9)	78.4 (73.4,82.4)	9.7 (6.5,14.2)
**Chronic count**				
0	18.9 (17.6,20.3)	23.1 (20.7,25.5)	73.0 (70.5,75.3)	4.0 (3.2,5.0)
1	41.0 (38.5,43.5)	12.1 (10.5,13.9)	81.4 (79.4,83.3)	6.5 (5.1,8.1)
2+	59.4 (55.7,63.0)	5.7 (4.6,7.2)	77.2 (73.7,78.4)	17.0 (14.2,20.4)

^a^Confidence Interval

^b^Completed

^c^University

^d^Divorced

Arthritis, Depression, Asthma measured using WHO algorithms. Diabetes measured using self-report.

In Table 3, the proportion of respondents with low intensity (n = 1,311) vs. moderate intensity (n = 6,804) vs. high intensity (n = 700) back pain in the pooled sample is shown by socio-demographic and health characteristics. There were more females than males were in the high intensity group (9% vs. 6%). About 10 to 13% of respondents who did not complete primary education, were separated, divorced or widowed, not working, operated at low levels of physical activity, and had diabetes, were in the high intensity group. Over 20% of respondents with depression, 18% with asthma, and 17% with two or more chronic conditions were in the high intensity group. An age gradient is evident for the high intensity group; 21% of respondents aged 80 and over had high intensity back pain compared with 5% of respondents aged 50 to 59 years.

Tables 4, 5 and 6 present the results of the adjusted multivariable regressions. Reference categories for the education, employment, wealth status and physical activity variables were changed from those shown in Tables 1 and 3 in order to show odds as risk.

**Table 4:** Multivariable logistic regression of factors associated with back pain prevalence, adults 50+ years, pooled, SAGE Wave 1 (N = 30,146).

	Adjusted Odds Ratio	95% CI^a^
**Sex (Reference: male)**		
Female	1.6***	1.4,1.8
**Age Group (Reference: 50–59 years)**		
60–69 years	1.1	1.0,1.2
70–79 years	1.1	1.0,1.3
80+ years	1.0	0.8,1.2
**Education (Reference: completed university/college)**		
Completed secondary/high	1.4**	1.1,1.7
Completed primary	1.6**	1.1,2.1
No primary completed	2.0***	1.5,2.5
**Marital Status (Reference: never married)**		
Married/cohabiting	1.6**	1.1,2.2
Separated/divorced/widowed	1.5**	1.1,2.2
**Work Status (Reference: currently working)**		
Never worked	1.0	0.9,1.2
Not currently working	1.1	1.0,1.2
**Wealth Status (Reference: highest-wealthiest)**		
Second highest	1.1	0.9,1.3
Mid	1.2	1.0,1.5
Second lowest	1.2	1.0,1.4
Lowest (poorest)	1.4**	1.1,1.7
**Residence (Reference: urban)**		
Rural	1.2**	1.0,1.4
**Smoker (Reference: not current)**		
Current	1.2*	1.0,1.3
**Alcohol (Reference: never drinkers)**		
Former drinkers	1.2	1.0,1.4
Current drinkers	1.2**	1.1,1.5
**Waist Circumference (Reference: low risk)**		
High risk	1.1	0.9,1.2
**Physical Activity (Reference: low)**		
Moderate	1.0	0.9,1.2
High	1.1	1.0,1.3
**Chronic Conditions Count (Reference: none)**		
1	2.7***	2.4,3.0
2+	4.8***	4.1,5.6
**Country (Reference: China)**		
Ghana	2.2***	1.9,2.6
India	1.8***	1.5,2.2
Mexico	1.8**	1.2,2.8
Russian Federation	4.1***	3.1,5.4
South Africa	2.3***	1.9,2.8

^a^Confidence Interval

*** <0.01

**p<0.05

*p<0.1

Variance Inflation Factor = 1.17.

**Table 5:** Multivariable ordinal logistic regression of factors associated with back pain intensity, adults 50+ years, pooled, SAGE Wave 1 (N = 8,815).

	Adjusted Odds Ratio	95% CI^a^
**Sex (Reference: male)**		
Female	1.3**	1.1,1.6
**Age Group (Reference: 50–59 years)**		
60–69 years	1.3**	1.1,1.6
70–79 years	1.7***	1.3,2.2
80+ years	3.3***	2.2,4.9
**Education (Reference: completed university/college)**		
Completed secondary/high	1.2	0.8,1.7
Completed primary	1.4	0.9,2.2
No primary completed	1.6**	1.0,2.6
**Marital Status (Reference: never married)**		
Married/cohabiting	1.1	0.7,1.7
Separated/divorced/widowed	1.1	0.6,1.7
**Work Status (Reference: currently working)**		
Never worked	1.2	0.9,1.6
Not currently working	1.3**	1.0,1.5
**Wealth Status (Reference: highest- wealthiest)**		
Second highest	1.0	0.7,1.3
Mid	1.2	0.9,1.6
Second lowest	1.2	0.9,1.6
Lowest (poorest)	1.2	0.9,1.6
**Residence (Reference: urban)**		
Rural	1.6***	1.3,2.0
**Smoker (Reference: not current)**		
Current	1.2**	1.0,1.5
**Alcohol (Reference: never drinkers)**		
Former drinkers	1.1	0.9,1.3
Current drinkers	1.2	0.9,1.4
**Waist Circumference (Reference: low risk)**	1.2	1.0,1.4
High risk	1.2	1.0,1.4
**Physical Activity (Reference: low)**		
Moderate	0.8	0.7,1.0
High	0.8*	0.6,1.0
**Chronic Conditions Count (Reference: none)**		
1	1.9***	1.6,2.2
2+	4.2***	3.4,5.3
**Country (Reference: China)**		
Ghana	2.0***	1.5,2.7
India	2.0***	1.5,2.7
Mexico	1.0	0.6,1.6
Russian Federation	1.2	0.8,1.8
South Africa	1.1	0.8,1.6

^a^Confidence Interval

*** <0.01

**<0.05

*p<0.1

Variance Inflation Factor VIF = 1.15.

**Table 6:** Multivariable regression of factors associated with disability, adults 50+ years, pooled countries, SAGE Wave 1 (N = 29,996).

	Adjusted Coefficient	95% CI^a^
**Back Pain Intensity (Reference: No back pain)**	****	****
Low	-3.3***	-4.2,-2.5
Moderate	5.0 ***	4.2,5.7
High	18.6***	16.5,20.6
**Sex (Reference: males)**	****	****
Female	0.9**	0.3,1.5
**Age Group (Reference: 50–59 years)**		
60–69 years	1.4***	0.8,1.9
70–79 years	5.4***	4.6,6.2
80+ years	13.0***	11.3,14.7
**Education (Reference: completed university/college)**		
Completed secondary	2.8***	1.8,3.9
Completed primary	4.2***	3.1,5.2
No primary	5.4***	4.4,6.5
**Marital Status(Reference: never married)**		
Married	-1.4	-3.7,0.9
Separated/divorced/widowed	-0.1	-2.2,2.6
**Work Status (Reference: currently working)**		
Never worked	3.9***	2.8,5.0
Not currently working	3.1***	2.3,3.8
**Wealth Status (Reference: highest)**		
Second highest	1.4***	0.9,2.4
Mid	2.7***	1.7,3.8
Second lowest	3.3***	2.3,4.2
Lowest (poorest)	4.8***	3.9,5.8
**Residence (Reference: urban)**		
Rural	3.0***	2.1,3.9
**Smoker (Reference: not current)**		
Current	-0.2	-0.8,0.5
**Alcohol (Reference: Never drinkers)**		
Former drinkers	0.7	-0.2,1.6
Current drinkers	-0,4	-1.0,0.3
**Waist Circumference (Reference: low risk)**		
High risk	-0.3	-0.0,0.3
**Physical Activity (Reference: low)**		
Moderate	-3.9***	-4.7,-3.0
High	-4.9***	-5.7,-4.1
**Chronic Conditions Count (Reference: none)**		
1	3.8***	3.1,4.4
2+	9.3***	8.3,10.3
**Country (Reference: China)**		
Ghana	10.3***	9.1,11.4
India	15.3***	14.3,16.3
Mexico	4.4***	2.1,6.8
Russian Federation	8.1***	6.5,9.7
South Africa	7.9***	6.7,9.1

^a^Confidence Interval

***p<0.01

**<0.05

*p<0.1

Variance Inflation Factor VIF = 2.05.

In Table 4, female sex, rural residence, being married or separated/divorced/widowed compared with never being married, being a current smoker, and drinking alcohol, were statistically significantly in association with back pain. There is a gradient in the association between education level and back pain. Respondents who had not completed even primary education had almost two-fold higher odds of reporting back pain compared with those who completed university/college. The odds of back pain were 40% higher for respondents in the lowest (poorest socioeconomic quintile) compared with respondents in the highest quintile (as the reference group). The odds of back pain increased with the number of chronic comorbidities, (i.e. from 2.7 times for those with one condition, to 4.8 times for those with two or more comorbidities). There was no statistically significant association between back pain and age, physical activity level, waist circumference and employment status. Country odds ratios, with China as the reference, were statistically significant. Adults in the Russian Federation, for example, had four-fold higher odds of reporting back pain compared with adults in China.

In Table 5, the ordinal logistic regression presents the odds of reporting high intensity back pain vs. low intensity back pain and high intensity back vs. moderate intensity back pain for each of the covariates. Older age, female sex, living in a rural area, not completing primary education, not currently working, being a current smoker, and having multiple chronic conditions, were statistically significant in association with higher back pain intensity. A clear gradient is seen across age, with individuals aged 80 and over having three times higher odds of high back pain intensity compared with those aged 50 to 59 years. Unlike the results in Table 4, marital status and wealth were not statistically significant in association with high back pain intensity.

In Table 6, the multivariable linear regression shows association between levels of back pain intensity and the continuous WHO disability score. The reference group comprises respondents with no back pain, according to self-reported past-month back pain prevalence. Compared with the non-prevalent reference group, people in the high intensity group had, on average, a 19-unit worse disability score, and people in the lowest intensity group had less disability, when all other variables were held constant. The disability score for respondents in the moderate disability group was, on average, five units higher than the score for those without reported back pain after adjusting for all other variables.

The sample size (N = 29,996) is due to missing data (n = 150) on the intensity score for respondents who were included in prevalence estimates.

Disability was also associated with socioeconomic factors and comorbidities when back pain intensity was held constant. There are inverse associations between education, wealth and disability and there was positive association between disability and comorbid chronic conditions. There was higher disability amongst the oldest age group and rural residents. People with higher physical activity had less disability compared with those with low physical activity, and those who never worked had higher disability compared to those who were working. Compared with China, adults aged 50 and older in the other five SAGE countries, had more disability.

## Discussion

This study of the six SAGE countries is the first to utilize nationally representative, comparable, population survey data to measure and assess factors associated with past-month back pain prevalence across six culturally different LMICs. These findings are a start but not sufficient. They serve as a reference point for clinicians, public health practitioners and researchers planning future qualitative and quantitative studies that can inform development of country-specific medical education and practice guidelines.

In recent years, there has been increasing recognition of the growing burden of musculoskeletal disease and back pain in both developed and developing countries [5, 8, 9, 11, 43]. A large multi-country study of chronic pain conditions [42] showed that the age standardized prevalence of chronic pain conditions in the previous twelve months was 37.3% in developed compared with 41.1% in developing countries, with back pain more common in developing countries.

Across the six SAGE countries, past-month back pain prevalence was almost 30%. These results are within range of prevalence estimates reported in some other studies. Estimates of one-month back pain prevalence for adults aged 60 and over in developing countries range between 18% and 29% (13). In our study prevalence estimates varied across the SAGE countries, from 22% in China to 56% in the Russian Federation. The estimates for the two African countries, Ghana and South Africa, were 41% and 39% respectively. A review of back pain prevalence studies in adults aged 20 to 85 years in the African continent reported one-year back pain prevalence between 40% and 72% [25]. The high back pain prevalence seen here in the Russian Federation is consistent with a previous analysis of World Health Survey data in which the prevalence was 76.8% in the major metropolitan areas of Moscow and St Petersburg [59].

In addition to pharmacological interventions, treatment and management modalities for back pain include behaviour and exercise therapy and lifestyle change, many of which are relatively low-cost to implement in primary care settings [60]. However, most of what is known and demonstrated comes from developed countries. Context-specific trials and evaluations in developing countries are needed.

In agreement with other studies [15, 16, 61] this study shows that back pain increases with age although not necessarily for the very old, and that female sex is significantly associated with back pain [16, 18, 24]. The reasons for this are not clear, although it is suggested that this may be due to greater sensitization to pain, the reporting of pain, and differences in response to analgesics in females [42, 62, 63]. Even though the mechanisms that lead to gender differences in pain are yet to be elucidated, in their literature review, Bartley and Fillingim [63] suggested that multiple bio-psychosocial mechanisms (e.g. genetic, sex hormones, pain coping, gender roles) may interact and contribute to the phenomenon.

Other studies have also reported inverse socioeconomic gradients between the prevalence and intensity of back pain and education and wealth [14, 32, 35]. Our study also found that people living in rural areas were more likely to experience back pain and at higher intensity. This may have also been due to more frequent and strenuous outdoor household activities (e.g. carrying water or food), undertaken by older people living in rural areas in these six countries [14, 36, 37]. The occupational variable in SAGE that identified physical labour however had considerable missing data, and for that reason, was not included here. Instead, we used a measure of working status for which the data were over 98% complete. However, a simple cross tabulation in the pooled SAGE dataset showed that over 51% of rural residents were in occupations involving physical labour compared to 38% of urban residents.

There was a higher prevalence of back pain among respondents with high risk waist circumferences, although in the presence of other factors in the multivariable models, waist circumference was not statistically significant. The precision of these estimates is influenced by missing data in the waist circumference variable.

In this study of the six SAGE countries there was a higher prevalence of back pain amongst those with high levels of physical activity and the odds of reporting back pain were slightly higher for those who had high, compared with moderate or low levels of physical activity. However, these results were not statistically significant and the association between back pain and physical activity can occur in both directions. For example Kim et al. [64] found that vigorous and moderate physical activity in older Koreans was associated with an increased risk of back pain in both men and women, whereas strength exercises were associated with a reduced risk of back pain.

In the pooled analysis of adults aged 50-plus in the SAGE countries, about 8% of those with back pain experienced it at high intensity, although at the country level this ranged from 4% in China to 12% in India. Respondents who experienced high intensity back pain had considerably greater disability, compared with those with low intensity or no back pain. Musculoskeletal disorders are a frequent cause of disability in older populations [40] and these findings reflect the major disabling effect of back pain on daily function and activities. In the multivariable disability model, the effect of female sex was not evident as in the other models, but there was a clear age gradient, with older age significantly associated with greater disability. These results are generally consistent with other studies. In a study of Korean adults with a mean age of 40 years, Kim et al. [64] found that the degree of disability from back pain assessed was influenced by a pain severity and type. In the US Weiner et al. [65] showed that, in a large cohort of well-functioning adults aged 70–79 years, back pain frequency/intensity was associated with perceived difficulty in performing important physical functional tasks. These authors suggested that the dose-response relationship between back pain frequency/intensity and self-reported functional task difficulties underscores the importance of efforts to treat and reduce pain without necessarily eradicating it [65].

The WHO measure of disability [48] takes into account variations in the reporting of disability across cultures [66]. There is increasing evidence of the effectiveness of low-cost easily implemented therapeutic interventions, such as physical exercise, in improving rehabilitation outcomes for people with back pain as well as the quality of life for people with disabilities [67] in higher income countries. The finding of strong association between back pain and disability in the SAGE countries has important public health policy implications for LMICs. One of the reasons for this is that the data were population-based being captured in households, rather than in clinical settings or the workplace. This suggests the need for investment in community-based primary care assessment and education.

### Limitations

The cross sectional nature of the study presents limitations in terms of interpreting causal association. We cannot separate antecedent factors that influence incident cases from consequent factors associated with prevalent cases. Some determinants may also be consequences, e.g. smoking and drinking, and there may also be selection effects, e.g. those with back pain are unable to undertake physical activity at high levels but physical activity may also be a causal factor. Data from future waves of SAGE will provide information about the direction of associations.

Although the GPAQ was the best available measure of physical activities in this dataset, it is possible that we underestimated the amount of physical activities undertaken by these older adults. The GPAQ only captures work and recreational activities and does not include indoor and outdoor household activity, which can be significant in developing countries, particularly in rural areas.

The WHODAS 2.0 disability score captures respondents that have difficulties in performing everyday tasks that may not be attributable to back pain. Nevertheless, the WHODAS 2.0 was the most appropriate measure of disability for this study.

We developed an index for back pain intensity and frequency using questions that referred to pain in general, rather than back pain specifically. While it is true that the pain that the individual reports could have been due to a number of conditions, we assume that back pain was one of these. Therefore if back pain was experienced in the previous 30 days, we assume that it would have contributed to the responses to the questions about general pain that were used to develop the intensity/frequency index.

A large proportion of the data in the SAGE were self-reported, and could be influenced by the reporting heterogeneity by the respondents, either due to their experiences or expectations. Salomon et al. [68] suggested the use of anchoring vignettes based on fixed levels of health on different dimensions such as mobility, pain, cognition, to adjust for this. However Hirve et al. [69] analysed eight health and demographic surveillance sites within the WHO-INDEPTH Network and found that the use of vignettes to adjust for reporting heterogeneity could not be justified because vignette equivalence and response consistency requirements were not fulfilled.

Because there was a relatively low percentage of missing data, we decided against using multiple imputation methods. We acknowledge the possibility of bias due to missing data.

### Strengths

Measures of back pain prevalence are typically based on self-report. Estimates vary widely across populations and settings owing to methodological, definitional and socio-cultural differences [11, 12, 36]. Socio-cultural and psychosocial factors influence the reporting of pain, as well as knowledge and perception of ways of dealing with the impact of pain on everyday functioning [1, 41]. The SAGE adjusted for cultural differences as far as possible by using standardized culturally appropriate instruments.

This is the first study of its kind to use nationally representative standardized population survey data to present detailed contextual analyses of back pain and disability in older adults in LMICs. The questionnaire was first translated into the local language, and then back translated. All translations were validated before data collection commenced. The interviews were administered by trained local interviewers in face-to-face, one-on-one settings to ensure cultural appropriateness.

The study reveals differences between countries that can be followed up by policy makers. For example, the high back pain prevalence in the Russian Federation contrasts with low back pain prevalence in China. Amongst those who experienced back pain, a relatively high proportion of respondents in India reported high intensity back pain, while a high proportion reported low intensity back pain in Mexico, and a high proportion reported moderate intensity back pain in South Africa. Ghana and India had greater disability compared with China. Although the SAGE questionnaires are designed to accommodate cultural differences, there may be other reasons for the between-country differences. This is certainly an area for further research.

## Conclusions

Our study highlights the need to further gather data and investigate back pain determinants in older adults within country settings [5]. We do not yet fully understand the impact of sociocultural factors on the perception and reporting of back pain in developing countries [29]. Our findings are a start but certainly not sufficient. What is needed is context-specific data that can inform the design, development, trialling and economic evaluation of interventions for the diagnosis, management, treatment and rehabilitation of back pain in older adults in developing countries.
